# Comorbid Conditions, Mental Health and Cognitive Functions in Adults with Fibromyalgia

**DOI:** 10.1177/0193945920937429

**Published:** 2020-06-26

**Authors:** Sophie Taylor, Penny Furness, Simon Ashe, Sarah Haywood-Small, Kim Lawson

**Affiliations:** 1 Sheffield Hallam University, College of Social Sciences and Arts, Sheffield, UK; 2 Sheffield Hallam University, College of Health, Wellbeing and Life Sciences, Sheffield, UK

**Keywords:** fibromyalgia, adulthood, comorbid conditions, mental health

## Abstract

This study examined age group differences across adulthood in comorbid conditions, mental health, and cognitive function in people with fibromyalgia. Participants completed an online survey about how fibromyalgia affects their everyday life. Chi square analyses were conducted to examine associations between age groups and (a) comorbid conditions and (b) severity of anxiety and depression. ANOVA analyses examined age group differences on aspects of self-report cognitive function. The greatest prevalence of comorbid conditions was found in middle adulthood. Early adulthood was associated with more cases of severe anxiety with the lowest number of cases being in the oldest age group. Middle adulthood was associated with worse self-report pain compared to the youngest age group. Older adults showed better self-report cognitive function compared to younger adults. Distinct age profiles based on comorbid conditions, mental health, and symptom severity across adulthood in fibromyalgia have been demonstrated.

Fibromyalgia is a chronic condition associated with pain, disturbed sleep, fatigue, cognitive impairment, depression, and anxiety (Lawson, 2017). The prevalence of fibromyalgia varies across adulthood. In the US population, the prevalence of fibromyalgia was reported as being the lowest in the 18–29 years group and highest in the 50–59 years group (Walitt et al., 2015). Fibromyalgia is often diagnosed in middle adulthood (Cronan et al., 2002), although there are reports of juvenile fibromyalgia (Kashikar-Zuck et al., 2016). Chronic pain in late adolescence and early adulthood could negatively impact education and employment, independence, and peer and romantic relationships (Rosenbloom et al., 2017). Patients in early adulthood who expect to have an active lifestyle may perceive a chronic health condition as more disruptive compared to someone in later adulthood (Cronan et al., 2002). Thus, the condition may have different effects on quality of life depending on the life stage of an individual.

Historically, health has been viewed using the biomedical model. A criticism of the medical model is that psychological and social factors are neglected, with these factors being included in the biopsychosocial model (Engel, 1980). More recently, Lehman and colleagues (2017) proposed the dynamic biopsychosocial model that incorporates biological, psychological, interpersonal, and contextual factors affecting health. A key aspect of this model is that the factors interact over time. The dynamic biopsychosocial model includes notions from Bronfenbrenner’s ecological model (1986) where the microsystem, mesosystem and exosystem influence health. The dynamic biopsychosocial model applies the lifespan approach to health and indicates that different factors may be more important at certain developmental stages (Lehman et al., 2017).

Some studies have examined age group differences in fibromyalgia symptoms, mental health, and quality of life. Cronan et al. (2002) compared young (20–39 years), middle-aged (40–59 years), and older adults (60–85 years) on self-reported pain and the effects of fibromyalgia on everyday life, sleep, depression, and well-being. Participants in the older group reported significantly less pain, depression and impact of illness, and better sleep quality compared to younger and middle-aged groups. These results may be explained by older adults attributing fibromyalgia symptoms to normal aging (Jiao et al., 2014). There were no age-group differences in psychosocial variables of coping styles, self-efficacy, and helplessness, indicating that these did not influence fibromyalgia improvements with age. A limitation is that the direction of relationship between mood and health could not be ascertained in the cross-sectional design of the study (Cronan et al., 2002). Another study found that younger (≤39 years) and middle-aged groups (40–59 years) reported poorer symptom effects on daily functioning and poorer quality of life compared to the older group of those aged 60 years and over (Jiao et al., 2014).

Contrasting results were reported in a study about anxiety, depression and alexithymia in fibromyalgia patients in early (≤ 35 years), middle (>35 and <65 years) and older adulthood (≥65 years). The younger age group reported significantly lower anxiety, depression, and alexithymia scores compared to the oldest age group, indicating that early adulthood was associated with more optimal functioning relative to older adulthood (Peñacoba Puente et al., 2013). Therefore, previous research has found contrasting results in terms of age group differences in fibromyalgia. Inconsistent findings may be due to studies having different cut-off ages for early, middle, and older adulthood. Furthermore, participants’ symptom severity and time since diagnosis may vary between studies.

Dyscognition, impaired cognitive function in fibromyalgia, is part of a recently developed multi-dimensional diagnostic framework (Arnold et al., 2019). Moriarty et al. (2011) proposed a model of pain-related cognitive impairment based on pain leading to limited cognitive resources, altering neuroplasticity and dysregulating neurochemistry. There is convergent evidence of dyscognition from performance-based (Muñoz Ladrón de Guevara et al., 2018) and subjective measures (Williams et al., 2011). A meta-analysis showed that fibromyalgia was related to poorer performance on cognitive function tasks compared to control groups (Bell et al., 2018). There were greatest group differences for inhibitory control, medium effect sizes for processing speed, short- and long-term memory and updating, with a smallest effect size for set shifting. Existing research has showed executive function impairments in fibromyalgia in adults aged 50 years and over (Cherry et al., 2014), but there is a lack of research on fibromyalgia and cognitive function across the whole adult lifespan.

Previous research indicates the presence of comorbid conditions in fibromyalgia. A study examined hospital records in Turkey and found that 67.8% of the sample of patients with fibromyalgia were diagnosed with one or more comorbid conditions, with cardiovascular, endocrine, and mental disorders being the most prevalent (Bilge et al., 2018). Häuser et al., (2018) suggested a treatment approach based on subgroups characterized by pain, mental health, and comorbid conditions. Therefore, it is important to ascertain comorbid conditions affecting patients with fibromyalgia so that awareness may inform diagnosis and treatment options.

## Purpose

The present study examined self-report comorbid conditions, fibromyalgia symptoms, and cognitive function in participants with fibromyalgia across the adult lifespan. Previous studies have split adulthood into early, middle, and late adulthood. The present study extends previous research by analyzing group differences in more fine-grained age groups.

## Method

A qualitative interview study (Ashe et al., 2017) informed the development of the online survey. The survey assessed symptom severity, mood, cognitive impairment, and comorbid conditions because these have not previously been assessed across adulthood in people with fibromyalgia using fine-grained age groups. Research indicates that fibromyalgia is associated with depression, anxiety, and cognitive impairment (Bell et al., 2018) so pre-existing measures of symptom severity, mood and cognitive impairment were included in the survey. Fibromyalgia is associated with comorbid conditions (Bilge et al., 2018), although previous research has not always assessed these, and comorbid conditions could influence treatment success. The survey was advertised from January to April 2016 by fibromyalgia charities, networks and support groups. Using data from the online survey we have published a qualitative paper about perceived causes of fibromyalgia (Furness et al., 2018), a quantitative study about the effectiveness of pharmacological and nonpharmacological treatments (Taylor et al., 2019), and a qualitative paper about attitudes to physiotherapy (Furness et al., 2019). This research received ethical approval from Sheffield Hallam University Research Ethics Committee (FREC/SW/125-FUR). Completion and submission of the online survey was taken as implied consent.

### Design

This study used a cross-sectional design to compare comorbidities, mental health, and symptom severity across six age groups in adulthood.

### Participants

Inclusion criteria for participants were to be aged 18 years or over with an official diagnosis of fibromyalgia. Nine hundred and forty-one participants aged between 18 years and 87 years (*M* = 47.70, *SD* = 12.09) completed the online survey.

### Measures

The online survey included the following measures:

***Fibromyalgia symptoms*** Participants completed the “symptoms of sensory reactivity” and “symptoms of pain and consequences” scales from the Comprehensive Rating Scale for Fibromyalgia (CRSFS; López-Pousa et al., 2013). Participants considered symptoms over the previous two weeks and selected a response from the following options: never/once, several days, more than half of the days, and almost every day. There were six items assessing sensory reactivity symptoms (e.g., joint discomfort that has restricted movement), and seven items assessing pain and consequences (e.g., general tiredness that has prevented you from performing your daily activities). Possible scores ranged between 0 and 54 for the sensory reactivity subscale, and between 0 and 63 for the pain and consequences subscale. Higher scores indicated greater sensory reactivity and consequences of pain. The subscales showed good reliability (Cronbach’s α for sensory reactivity = 0.89 and Cronbach’s α for pain and consequences = 0.87).

***Anxiety and depression*** Participants completed the Hospital Anxiety and Depression Scale (HADS; Zigmond & Snaith, 1983) by selecting a response from four options about how they had felt in the past week. Seven items assessed anxiety (e.g., worrying thoughts go through my mind) and seven items assessed depression (e.g., I look forward with enjoyment to things). Possible scores were between 0 and 21, with scores categorized as normal (0–7), mild (8–10), moderate (11–15), and severe (16–21; Snaith & Zigmond, 1994). The separate scales were reliable (Cronbach’s α anxiety = 0.84 and Cronbach’s α depression = 0.83).

***Cognitive impairment*** Participants completed the Multidimensional Inventory of Subjective Cognitive Impairment (MISCI; Kratz et al., 2015) by rating 10 statements using a five-point Likert scale about their cognitive function in the previous seven days. Two items assessed mental clarity, memory, attention, executive function, and language. An example of a mental clarity item is ‘I have been able to think clearly without extra effort.’ Possible scores ranged from 10 to 50, with higher scores indicating better self-report cognitive function and lower impairment. The reliability (Cronbach’s α = 0.66) was below the acceptable value of 0.7 that Nunnally (1978) recommended.

**Self-report comorbid conditions** Participants selected whether they had experienced any of the following conditions: chronic fatigue, arthritis, irritable bowel syndrome (IBS), thyroid, diabetes, circulation disorders, coeliac disease, migraine, mitochondrial dysfunction, hypermobility, anxiety, and depression.

### Data Analysis

Chi square analyses examined associations between age groups and the presence of comorbid conditions. Further chi square analyses were conducted to examine associations between age group and classification of anxiety and depression (normal, mild, moderate, and severe). ANOVAs were conducted on MISCI total scores and subscale scores to examine whether age group had a significant effect on cognitive function. If the ANOVA was significant then pairwise comparisons with Bonferroni adjustment were conducted. ANOVAs were conducted on CRSFS sensory reactivity and pain, and consequences scores to examine whether age group had a significant effect on symptom severity.

## Results

### Sample Characteristics

The sample was divided into the age groups: 18–29 years (*n* = 80), 30–39 years (*n* = 136), 40–49 years (*n* = 277), 50–59 years (*n* = 309), 60–69 years (*n* = 109), and 70–87 years (*n* = 27). Three participants did not report age data and were excluded from analyses. Eight hundred and ninety-two female participants and forty-five male participants completed the survey, with three participants not reporting gender and one participant identifying as mixed gender. Nine hundred and two participants reported ethnicity as white, fifteen participants reported mixed ethnicity, followed by nine participants identifying as Asian and six as black. One participant reported being from the following ethnicities: Greek, Irish / Greek Cypriot, Middle Eastern, and Portuguese. Four participants did not report ethnicity.

### Comorbid Conditions by Age Group

Table 1 shows frequencies and chi square results for associations between comorbid conditions and age groups. There was an association between age groups and the presence of arthritis, with the highest prevalence in the 50–59 years age group and lowest prevalence in the 18–29 years age group. There was also an association between thyroid comorbidity and age groups. Similarly, the highest prevalence of thyroid conditions was in the 50–59 years age group whereas the lowest prevalence was in the oldest age group. Chi square results showed an association between age groups and diabetes, with the 50–59 years age group reporting the highest prevalence. There were fewer than five reports of diabetes in the youngest and oldest age groups. The association between age groups and migraine was significant, with the 60–69 years age group reporting the most migraines and the oldest age group reporting the fewest migraines. Chi square results showed an association between age groups and comorbid hypermobility, with the highest prevalence in the 50–59 year age group and lowest prevalence in the oldest age group. There was no association between age groups and the presence of IBS, circulation disorders, coeliac disease, mitochondrial dysfunction, or chronic fatigue.

**Table 1.** Comorbid Conditions by Age Group.

Condition	18–29 years n (%)	30–39 years n (%)	40–49 years n (%)	50–59 years n (%)	60–69 years n (%)	70–87 years n (%)	χ^2^
Irritable Bowel Syndrome	52.0 (5.5)	98.0 (10.4)	202.0 (21.5)	223.0 (23.8)	84.0 (9.0)	13.0 (1.4)	10.93
Arthritis	9.0 (1.0)	33.0 (3.5)	104.0 (11.1)	169.0 (18.0)	67.0 (7.1)	17.0 (1.8)	92.71**
Circulation disorders	28.0 (3.0)	39.0 (4.2)	92.0 (9.8)	101.0 (10.8)	33.0 (3.5)	6.0 (0.6)	2.61
Thyroid	5.0 (0.5)	18.0 (1.9)	49.0 (5.2)	78.0 (8.3)	26.0 (2.8)	7.0 (0.7)	21.44**
Coeliac	6.0 (0.6)	< 5.0	11.0 (1.2)	15.0 (1.6)	< 5.0	< 5.0	4.64
Diabetes	< 5.0	6.0 (0.6)	22.0 (2.3)	36.0 (3.8)	9.0 (1.0)	< 5.0	15.47**
Migraine	50.0 (5.3)	83.0 (8.8)	190.0 (20.3)	172.0 (18.3)	59.0 (6.3)	6.0 (0.6)	28.73**
Hypermobility	31.0 (3.3)	37.0 (3.9)	45.0 (4.8)	56.0 (6.0)	17.0 (1.8)	< 5.0	30.99**
Mitochondrial dysfunction	< 5.0	< 5.0	8.0 (0.9)	6.0 (0.6)	< 5.0	< 5.0	2.84
Chronic fatigue	59.0 (6.3)	103.0 (11.0)	210.0 (22.4)	232.0 (24.7)	78.0 (8.3)	15.0 (1.6)	5.99

* p < 0.05, **p < 0.01.

Overall, the comorbidity data indicates higher reports of comorbid conditions in the 50–59 years group for arthritis, thyroid, diabetes and hypermobility and for migraines in the 60–69 years age group. The youngest age group reported the lowest prevalence of arthritis and diabetes. The oldest age group reported the lowest prevalence of thyroid conditions, diabetes, migraines, and hypermobility. This data suggests there are comorbid conditions associated with particular age groups across adulthood in fibromyalgia.

### HADS Anxiety and Depression Severity by Age Group

Table 2 shows frequencies of anxiety and depression severity by age group, and results from chi square analyses. There was an association between age group and HADS anxiety severity, but no association between age group and HADS depression severity. Around 43% of participants in the 18–29 years age group were in the severe anxiety category compared to the oldest age group where 15% of participants were in the severe anxiety category.

**Table 2.** Comorbid Anxiety and Depression Severity by Age Group.

Severity of condition	18–29 years n (%)	30–39 years n (%)	40–49 years n (%)	50–59 years n (%)	60–69 years n (%)	70–87 years n (%)	χ^2^
Anxiety Normal Mild Moderate Severe	10.0 (1.1) 16.0 (1.7) 20.0 (2.2) 34.0 (3.7)	17.0 (1.8) 23.0 (2.5) 44.0 (4.7) 50.0 (5.4)	53.0 (5.7) 61.0 (6.6) 90.0 (9.7) 69.0 (7.4)	60.0 (6.5) 73.0 (7.8) 103.0 (11.1) 72.0 (7.7)	30.0 (3.2) 25.0 (2.7) 33.0 (3.5) 21.0 (2.3)	11.0 (1.2) 6.0 (0.6) 5.0 (0.5) < 5.0	38.76**
Depression Normal Mild Moderate Severe	20.0 (2.2) 21.0 (2.3) 25.0 (2.7) 14.0 (1.5)	35.0 (3.8) 27.0 (2.9) 43.0 (4.6) 29.0 (3.1)	64.0 (6.9) 65.0 (7.0) 88.0 (9.5) 56.0 (6.0)	66.0 (7.1) 79.0 (8.5) 98.0 (10.5) 65.0 (7.0)	30.0 (3.2) 31.0 (3.3) 30.0 (3.2) 18.0 (1.9)	12.0 (1.3) 9.0 (1.0) < 5.0 < 5.0	17.04

** p < 0.01.

### Cognitive Function by Age Group

There was a significant effect of age on overall cognitive function, *F* (5, 913) = 10.00, *p* < 0.001, with the 70–87 years age group scoring significantly higher, indicating better cognitive function, compared to all other age groups (*p* < 0.01; Table 3). The 60–69 years group also scored significantly higher compared to the 30–39 years, 40–49 years and 50–59years groups (p ≤ 0.036). There was also a significant effect of age on mental clarity (*F* (5, 913) = 9.67, *p* < 0.001), with the oldest age group scoring significantly higher, indicating better mental clarity, compared to all younger age groups (*p* ≤ 0.01). Similarly, the 60–69 years group scored significantly higher, indicating better mental clarity, compared to younger age groups (p ≤ 0.037). Similarly, there was a significant effect of age on memory (*F* (5, 913) = 6.93, *p* < 0.001), with the oldest age group scored significantly higher, indicating better memory, compared to all younger groups (*p* < 0.01). ANOVA results showed a significant effect of age on attention, *F* (5, 913) = 9.58, *p* < 0.001. The 60–69 years group scored significantly higher, indicating better attention, relative to the 30–39 years, 40–49 years, 50–59 years, and 70–87 years year groups (all *p* < 0.05). The oldest age groups scored significantly higher compared to all other age groups (*p* < 0.01). There was no effect of age on executive function (*F* (5, 913) = 0.74, *p* = 0.597) or language (*F* (5, 913) = 0.78, *p* = 0.562).

**Table 3.** Descriptive Statistics for Total MISCI and Subscale Scores.

Aspects of cognitive function	18–29 years *n* = 76 *M* (*SD*)	30–39 years *n* = 133 *M* (*SD*)	40–49 years *n* = 270 *M* (*SD*)	50–59 years *n* = 305 *M* (*SD*)	60–69 years *n = 109* *M* (*SD*)	70–87 years *n* = 26 *M* (*SD*)	*F*
Total	23.42 (5.65)	23.32 (5.86)	23.34 (5.48)	23.66 (5.07)	25.50 (5.62)	30.19 (5.23)	10.00**
Mental clarity	4.09 (2.15)	4.18 (2.02)	4.17 (2.00)	4.37 (1.89)	5.05 (2.06)	6.54 (2.37)	9.67**
Memory	3.97 (2.03)	3.86 (2.05)	3.75 (1.92)	3.71 (1.78)	4.22 (2.03)	5.85 (1.83)	6.93**
Attention	3.96 (1.96)	3.86 (1.95)	3.92 (1.91)	3.97 (1.83)	4.67 (2.11)	6.23 (1.99)	9.58**
Executive function	5.74 (1.26)	5.71 (1.15)	5.73 (1.06)	5.85 (1.17)	5.84 (1.04)	5.58 (0.76)	0.74
Language	5.66 (0.92)	5.71 (0.79)	5.77 (0.80)	5.76 (0.83)	5.72 (0.89)	6.00 (0.80)	0.78

* p<0.05, **p < 0.01.

Overall, results indicate that older age groups performed better, compared to younger groups, on overall self-report cognitive function, mental clarity, memory, and attention. There was no effect of age on self-report executive function or language.

### Symptom severity

There was a significant effect of age on sensory reactivity scores (*F* (5, 900) = 3.42, *p* = 0.005; Table 4). The 40–49 years (*p* = 0.024) and 50–59 years (*p* < 0.01) groups scored significantly higher, indicating worse symptoms, compared to the 18–29 years age group. There was no effect of age group on pain and consequences scores (*F* (5, 884) = 1.46, *p* = 0.199).

**Table 4.** Descriptive Statistics for CRSFS Symptom Severity by Age Group.

CRSFS subscale	18–29 years *M* (*SD*)	30–39 years *M* (*SD*)	40–49 years *M* (*SD*)	50–59 years *M* (*SD*)	60–69 years *M* (*SD*)	70–87 years *M* (*SD*)	
	*n* = 75	*n* = 131	*n* = 268	*n* = 299	*n* = 107	*n* = 26	*F*
CRSFS sensory reactivity	32.28 (11.79)	34.90 (10.82)	36.58 (10.18)	37.06 (10.28)	34.64 (9.75)	34.69 (10.18)	3.42**
	*n* =74	*n* = 127	*n* = 263	*n* = 295	*n* = 105	*n* = 26	
CRSFS pain and consequences	39.59 (12.40)	41.61 (11.21)	41.90 (10.90)	41.72 (11.51)	40.50 (10.73)	36.92 (12.98)	1.46

** p < 0.01.

## Discussion

Comorbid conditions, mental health, and cognitive function across adulthood were examined in people with fibromyalgia. The results of this study indicate that distinct age profiles across adulthood exist in people with fibromyalgia. Early adulthood (18–29 years age group) was associated with the lowest prevalence of arthritis and diabetes, although the youngest age group had the lower pain and consequences scores and highest prevalence of severe anxiety compared to 40–49 years and 50–59 years groups. The 40–49 years and 50–59 years groups scored higher on pain and consequences compared to the youngest age group. The 50–59 years age group was also characterized by the highest reports of arthritis, thyroid, diabetes, and hypermobility. The 60–69 years age group was associated with the highest report of migraine and overall less impaired self-report cognitive function compared to the 30–39 years, 40–49 years, and 50–59 years age groups. The 60–69 years group reported less impaired mental clarity relative to younger groups and less impaired attention compared to 30–39 years, 40–49 years, 50–59 years and 70–87 years groups. The oldest age group (70–87 years) was characterized by the lowest prevalence of thyroid conditions, diabetes, migraine, and hypermobility. There were fewer reports of severe anxiety and less impaired overall cognitive function, mental clarity, memory, and attention in the oldest age group compared to younger groups.

These results support previous research showing that older adulthood is associated with better mental health and lower symptom severity in people with chronic pain or fibromyalgia (Cronan et al., 2002; Jiao et al., 2014; Wittink et al., 2006). A literature review found reports of high levels of resilience in some studies of older adults (MacLeod et al., 2016), and resilience was associated with lower health care utilization and improved self-rated health (Ezeamama et al., 2016). In a sample of people with fibromyalgia, resilience was found to increase positive affect and decrease negative affect, which in turn improved fibromyalgia symptoms (McAllister et al., 2015).

The finding of comorbid conditions, mental health and pain varying over the adult lifespan may be applied to the dynamic biopsychosocial model (Lehman et al., 2017). For example, greater pain and comorbid conditions being more often reported in middle adulthood compared to early adulthood aligns with the biological dynamic component of the model. Furthermore, the finding of severe anxiety being more prevalent in early adulthood and least prevalent in the oldest age group supports the psychological dynamics component of the model. The present analyses did not include interpersonal dynamics, and this aspect of the model is the most frequently omitted part (Suls & Rothman, 2004). Nevertheless, there is existing evidence of how biological, psychological, and social factors interact affecting health in chronic pain conditions including fibromyalgia (Adams & Turk, 2015; Furness et al., 2018).

There were significant associations between comorbid conditions and age groups in this sample of people with fibromyalgia. These data indicate that age 50–59 years in middle adulthood is a risk factor for poorer outcomes in terms of a higher prevalence of certain comorbid conditions, higher pain severity, and poorer self-report cognitive functions. Lifestyle factors, including poor diet, elevated body mass index, and smoking, may contribute to health conditions such as chronic widespread pain, and comorbid conditions of cancer and cardiovascular disease (VanDenKerkhof et al., 2011). The menopausal transition is also an important factor to consider in this predominantly female sample where the 50–59 years group was the largest group compared to other age groups. The average age of menopause has been reported as 51 years in the United Kingdom (Sarri et al., 2015), and menopausal transition has been associated with short-term cognitive function changes (Morgan et al., 2018). It is important to also treat comorbid conditions because this may improve quality of life (Bilge et al., 2018). NICE guidance indicates that HRT and cognitive behavioral therapy may assist with low mood or anxiety associated with the menopausal transition (Sarri et al., 2015), so these should be recommended if psychological symptoms of menopausal transition are a comorbid condition.

Fibromyalgia should be examined if a patient is presenting with pain and fatigue; if undiagnosed and untreated, comorbid fibromyalgia may result in incorrect treatment (Fitzcharles et al., 2018). The finding of distinct age profiles based on comorbid conditions, mental health, and severity of symptoms supports the conceptualization of subgroups in fibromyalgia. Häuser et al. (2018) suggested subgroups based on pain, mental health, and comorbidities, highlighting the importance of identifying the subgroups to enable individualized treatment approaches.

Age group comparisons of self-report cognitive function indicated that older age groups performed better, compared to younger groups, on overall self-report cognitive function, mental clarity, memory, and attention. There was no effect of age on self-report executive function or language. The assessment of executive function may be beneficial to younger adults with fibromyalgia because interventions may then be employed. Baker et al. (2017) identified several approaches aimed at improving cognitive function in chronic pain including psychoeducation, cognitive training, psychological therapy, medication optimization, and physical activity. The current study assessed self-report cognitive functions with the MISCI. Kratz et al. (2015) reported excellent internal consistency, although in the present study, internal consistency was below the acceptable threshold. In the present study if either ‘trouble planning the steps of a task’ or ‘harder than usual to express myself’ were deleted, this resulted in an acceptable reliability. Herreen and Zajac (2018) questioned the use of self-report cognitive function measures after finding that self-report cognitive function scores were better predicted by personality scores than performance-based cognitive function measures. It should also be noted that general fatigue was not assessed, and Williams et al. (2011) found that fatigue positively correlated with perceived cognitive dysfunction.

Future research should employ a sequential design examining trajectories across different age groups to assess developmental changes and identify treatments that may be beneficial at different stages of development (Peñacoba Puente et al., 2013). There is evidence of divergent trajectories with regards to pain and depression in participants with juvenile-onset fibromyalgia followed up after eight years (Black et al., 2019). Research using performance-based executive function tasks and including a mood assessment would further understanding of how affect and cognitive function are related in fibromyalgia.

In conclusion, the present study indicates age profiles based on comorbid conditions, mental health, cognitive functions and symptom severity across adulthood in fibromyalgia. Fibromyalgia assessment in the future should consider the developmental stage of people to enable identification of possible subgroups and the formulation of individualized treatment approaches.
